# But My Mother Is Beautiful: Understanding Body-Focused Anxiety Among Chinese Young Women as an Embodiment of Intergenerational Trauma

**DOI:** 10.1007/s11013-025-09946-z

**Published:** 2025-10-16

**Authors:** Hua Wu

**Affiliations:** University of Helsinki, Helsinki, Finland

**Keywords:** Body-focused anxiety, Embodiment, Intergenerational trauma, Chinese womanhood, Gendered experience, Mother–daughter relationship

## Abstract

Body-focused anxiety refers to a wide range of negative emotional and social experiences centered around discontent with one’s own body. This widely recognized phenomenon is mainly experienced by women across different contexts, often resulting from gendered socialization (Becker (1995) Body, self, and society: the view from Fiji. University of Pennsylvania Press, p. 2016). In modern Chinese society, it is important to understand these complex experiences within the quickly changing social landscape and ideas of womanhood. This study goes beyond clinical settings and the scope of mental illness (such as disordered eating or body dysmorphia) to include a variety of embodied experiences, including gender-related stress, negative self-awareness, and struggles in intimate relationships in the daily lives of young Chinese women. To grasp the current generation’s body-focused anxiety, this research suggests examining it through the lens of intergenerational trauma, particularly the embodiment of patriarchal injustice reflected in the deeply connected relationship between Chinese mothers and daughters. Over 7 years, this article presents person-centered ethnography to show that some of the most intense body-focused anxiety among young women is an intergenerationally shaped existential crisis rooted in their mothers’ upbringing within an explicit son-preference culture. As part of the intergenerational trauma, the body-focused anxiety is driven by (1) the mothers’ unresolved girlhood trauma of being “born into the wrong gender”; (2) the desire to fulfill social obligations, which reflects both intergenerational trauma and the gender-specific challenges faced in a changing patriarchy. This analysis emphasizes that body-focused anxiety is not just a reaction to current social pressures, but a reflection of identities shaped across generations. It reveals that “becoming women” in a specific cultural context is transhistorical and closely linked to unresolved traumas from previous generations. Manifesting in different forms, understanding young women’s body-focused anxiety from an intergenerational trauma perspective highlights how ongoing social forces are embodied even as cultural norms and social structures change.

## Introduction

Body-focused anxiety encompasses emotional processes, perceptions, beliefs, and behavioral patterns that adversely affect physical, social, and psychological well-being (Becker et al., 2002; Dittmar & Howard, 2004; Halliwell & Dittmar, 2004; Pidgeon et al., 2013). Predominantly affecting women, this anxiety often arises from dissatisfaction with physical appearance and an internalized pursuit of trendy feminine beauty ideals, such as the thin ideal or the “fit” body, perpetuated by mainstream media. Struggles with body-focused anxiety frequently lead to increased nervousness and discomfort in self-presentation (Becker et al., 2002; Dittmar & Howard, 2004; Tang et al., 2021). Consequently, distorted self-image and low self-esteem can significantly disrupt a woman’s daily routines, as well as her physical and mental health, social interactions, and ability to form meaningful relationships (Pidgeon et al., 2013). Unfortunately, compared to North American and Western European societies, the social and health burdens related to body-focused anxiety may be rising in some areas of Asia (Thomas et al., 2016). In China, concerns about being “fat,” body dissatisfaction, image sensitivity, and the desire to alter one’s body are no longer limited to the cognitive level but are more likely to translate into health-risking behaviors such as disordered eating, obsessive exercises, purging, and self-starvation. To make matters worse, the onset of such anxiety is occurring earlier in preadolescence (Thomas et al., 2016). Based on my observation, many young women who struggle with body-focused anxiety are involved in various forms of weight loss strategies that can damage their health. The most devastating and lasting health impact among these young women, driven by body-focused anxiety to lose weight and change their appearance, is the loss of body rhythm and the ability to interpret bodily signals. They struggle to feel hunger normally and cannot adjust their eating schedules according to appetite. Most of them also experience endocrine disorders and sleep disturbances, making it difficult to rest or sleep regularly when tired. Clearly, the health effects of body-focused anxiety extend far beyond eating disorders. Worse, their long-term practices of controlling diet, purging, taking various medications and supplements, gradually disconnect them from their bodies, leading to disrupted body rhythms.

While the damaging impact of body-focused anxiety has been widely acknowledged, it is crucial to gain a deeper understanding of the complex lived experiences of individuals grappling with such conditions, particularly regarding the origins of such anxiety (LaMarre & Rice, 2021). Current biomedical models in clinical settings primarily address the symptoms and dysfunctional behaviors associated with body-focused anxiety but fail to capture the intricate lived experiences that may not always manifest as symptoms, especially across diverse cultural contexts and social relations (Becker, 1995; Becker & Hamburg, 1996; Pidgeon et al., 2013). Additionally, the struggles outside clinical settings remain underexplored, with the social consequences of body-focused anxiety often treated as by-products of other psychological issues or overshadowed by the illness experience (Lester, 2019). I aim to incorporate the concept of intergenerational trauma, particularly the trauma transmitted through mother–daughter relationships, into the understanding of body-focused anxiety. In the Chinese context, the core of this trauma lies in the existential crisis experienced by undesired daughters who later become mothers of singleton daughters. Through the transition of identities and generational shifts, this existential crisis manifests in the shared experiences of two generations of women as the daughters “inherit” profound suffering, which manifest in their bodies as they become women.

I adopt a phenomenological approach to understanding the lived experiences of young Chinese women struggling with body-focused anxiety. In my ethnographic research on embodiment and emotion (Author, 2021), I observed that many young women exhibit body-focused anxiety, often engaging in behaviors that can harm their physical and mental health when faced with stress and uncertainty. Embodiment serves as a foundation for analyzing body–world interaction, enabling a broader interpretation that transcends observable events and embraces the dynamics of macro-level social changes while considering micro-level interactions (Csordas, 2002). Using this framework, I was able to notice a recurring theme for 7 years of person-centered ethnography: the first and foremost stress comes from their mothers. These women frequently referenced the traumatic experiences endured by their mothers, alongside their mothers’ preoccupation with their daughters’ bodies and behaviors, especially societal expectations of body image. This led me to hypothesize that intergenerational trauma plays a significant role in the manifestation of body-focused anxiety among these women, highlighting the complex interplay between familial experiences and individual emotional responses embedded in societal transformations.

I hereby define intergenerational trauma in this context as the struggle and suffering of the mothers’ generation due to existential denial and crisis, which is passed down to their daughters through emotional projection, lack of warmth, and mutual identity crises within the mother–daughter relationship. The origin of trauma originates with the mothers’ generation of women living under a patriarchal structure, shaped by misogynistic socialization and upbringing. By analyzing body-focused anxiety to expand the phenomenological experience of embodying intergenerational trauma, I explore various cognitive patterns, emotional experiences, illness perceptions, cultural practices, social interactions, and identity formation across different developmental stages. My ethnographic findings indicate that family members, especially mothers, play a crucial role in shaping young women’s perceptions of their bodies. Among the female participants in early adulthood (ages 19 to 25) who experience persistent body-focused anxiety and face developmental challenges, a common thread emerges: their mothers endured gender-based domestic violence during their girlhoods and were often regarded as unwanted daughters in families that prioritized sons (Guo et al., 2018; Li & Cooney, 1993; Wang et al., 2019). These mothers are more likely to project their existential struggles onto their daughters, fostering close emotional connections and engaging in body-related projects that reflect their battles against the objectification of the female body within a patriarchal culture. The mother–daughter relationship also displays similar attachment styles across case studies and personality types. They are fiercely loyal to each other and fiercely attached yet harbor intense dissatisfaction and frequently trigger each other during joint decisions. As one participant confessed, “I love my mother deeply. But she’s also the source of my issues, anxiety, and stress. I can’t help but share my life with her. At the same time, I want her out of my life so I can have peace.” On the “other side” of the narration, the mother expressed, “My daughter is the flesh of my body. She sometimes replicates experiences that scarred me deeply. I will do anything for her, and her resistance and reliance confuse me.”

Over the past 7 years, I carefully examined my participants’ narratives and my observations over 7 years, witnessing their transformation during emerging adulthood. By juxtaposing the manifestation of body-focused anxiety with the intergenerational experience of personal and historical transitions, I pose the following research questions:

1. What manifest symptoms and struggles do these participants experience, and how are they understood and handled by their mothers and themselves?
2. Contextualizing the underlying intention and desire, how and why do the participants trace these desires and discontent to their mothers’ experiences and early traumas?
3. Across a scope of 7 years, how did the mother–daughter relationships evolve?

I shall now integrate multiple lines of scholarship to provide a comprehensive understanding of body-focused anxiety among singleton daughters in contemporary China. Firstly, I examine the transformation of selfhood and social relations in Chinese society. I explore how social and familial structural transitions shifted people’s attention from being a filial child to becoming the committed parents of a competitive singleton in a neoliberal era. The situation can be especially tricky in a transforming patriarchy, when daughters had no pre-existing social position but are now being valued and evaluated as sons. Secondly, I will articulate how intergenerational trauma was originally formed and transmitted through the parent–child bond. In this context, the internalized trauma was deeply rooted in misogynistic culture and continued with gender-based bias and violence. Thirdly, I delve deeply into the hauntology of son-preference culture and analyze how young women are tasked with the impossible mission of overcoming their mothers’ existential crisis of not being born a son. This article highlights how body-focused anxiety can be understood through the lens of intergenerational trauma, particularly as it is transmitted in mother–daughter relationships. I hope to offer insights that extend beyond China and resonate with societies where rapid cultural transitions and strong patriarchal social norms continue to play a significant role.

## Aspiration and Anxiety in Contemporary China: Dynamics of Mother–Daughter Relationships in a Transforming Patriarchy

While previous research has explored how radical social changes impact selfhood and gender identity (Hsu & Madsen, 2019; Rofel, 1999; Yan, 2010; Yang, 2018; Zhang, 2019), there is a pressing need for a deeper analysis of traumatic experiences and their intergenerational effects. In the Chinese context, the relationship between mothers who grew up during the Collectivist era^1^ and their daughters who came of age under the One Child Policy^2^ creates a unique and complex dynamic (Fong, 2002; Greenhalgh, 2003). Against this back drop, the process of answering my research questions provides insight into the often-overlooked mother–daughter relationship in contemporary China and its impact on the well-being of both generations amidst their volatile bodies during precarious historical changes. As social norms evolve, the transition to womanhood presents unique challenges for each generation. Singleton young adult daughters (born between 1985 and 2000) directly embody the intergenerational trauma of their mothers, who were unwanted daughters subjected to negative treatment and unresolved existential crises as part of their gendered experiences. As women in the Market Era, the mothers’ generation regained social competence beyond their natal families and internalized new desires in the neoliberal age, which later transformed into intensified expectations placed upon their daughters. This dynamic is evident in the mothers’ enthusiasm and obsession with molding their daughters’ bodies throughout girlhood into newly desirable women.

Alongside the interview data, I was particularly intrigued by “unintentional slips” and behavioral patterns that contradicted individuals’ narratives. I have observed the driving forces that emerged from people’s subconscious into their behaviors and lifeworld (Sartre, 1970, pp. 151–152). In mother–daughter relationships characterized by embodied trauma, these driving forces represent conflicting emotions:

First, mothers encounter conflicting social demands and value systems. During their social integration as young adults in the 1990s, they received social recognition outside of their natal families for the first time. However, with the booming market economy and increased gender awareness in China, this quest for recognition encompassed not only the gender equality agenda that offers women non-domestic career options but also the perfectionist expectations of being a professional woman who embodies both gendered attractiveness and maternal achievements (Rofel, 1999; Kuan, 2005; Kajanus, 2005). Pursuing feminine perfection for their daughters also became a key focus in motherhood. Essentially, these women experienced abuse as girls, gained social recognition as career women, and felt unsupported as mothers of singleton daughters. At each stage of their life course, they face contradictory social standards that both suppress their gendered existence and demand their gendered performance.

The contrasting lives of singleton daughters and their mothers drive complex dynamics in their relationships. Singleton daughters, as only children, have access to abundant resources from birth and receive unconditional approval from grandparents, who once seemed abusive to the mothers. This situation fosters jealousy and resentment in mothers, making the mother–daughter relationship fraught with anxiety and projection. As singleton daughters grow into young women, a strategic collaboration often emerges between mothers and daughters, focusing on transforming the daughters’ bodies. This “body project” includes strict micromanagement, cosmetic surgery, weight loss programs, and corrections in posture, movements, routines, relationships, and self-perception. As daughters mature, these relationships often become filled with conflict and resistance from both sides. Understanding the turbulent personal and social history behind these dynamics is crucial for comprehending the complexities of the mother–daughter relationship.

The third driving force is the blurred boundary between mothers and daughters concerning their intimate social bond and their perceptions of their bodies. These two generations of women often view their bodies as a unity within the patriarchal family. Since bodies can embody cultural norms, desires, and power dynamics while being continually shaped by change (Grosz, 1994), mother–daughter dynamics can foster both deep cooperation and mutual rejection of their bodies. Mothers perceive their daughters’ bodies as extensions of their own—both biologically and emotionally—which inevitably projects their self-objectification or resentment onto their daughters’ developing forms. This establishes the foundation for the transmission of intergenerational trauma and, frequently, sexual repression.

To comprehend why gendered experiences and existential crises are transmitted to subsequent generations as body-focused anxiety, it is essential to contextualize this phenomenon within China’s rapidly changing social and familial structures. Following radical population policies and swift urbanization, the traditional patriarchal system in China, with its solid patrilineal foundation, has loosened within urban nuclear families (Greenhalgh, 2003; Kipnis, 2008; Santos & Harrell, 2017; Yan, 2003). Researchers have examined the mental health impacts of these transitions, noting that living amid uncertainty can heighten anxiety, widen generational gaps, and create emotional and ideological conflicts in close-knit nuclear families. This is particularly evident as moral crises and pressures in public life become privatized and psychologized (Hsu & Madsen, 2019; Kleinman et al., 2011; Yan, 2003; Yang, 2018; Zhang, 2014, 2019). Despite extensive analysis of the cross-generational and transhistorical trajectories of Chinese experiences, the intimate, emotionally charged, and complex relationships between mothers and daughters remain under-discussed (Evans, 2008, 2011; Evans & Strauss, 2010; Fong, 2002, 2004).

Harriet Evans’s exploration of mother–daughter relationships in urban China provides valuable insights into the complex dynamics that develop as mothers work to secure a prosperous future for their daughters amid changing cultural landscapes. Evans emphasizes the dual roles mothers serve as both nurturers and protectors, often dealing with anxiety and moral dilemmas while navigating rapidly shifting societal norms. The intergenerational anxiety and conflicts frequently center around their perceived and expressed sense of self as gendered individuals. Each generation creates its own victimhood narrative in response to historical changes, positioning itself in opposition to what they see as the “conservatism” of their mothers (Evans, 2008). In my research, I observe similar patterns among a younger group of daughters, who are about 10 years younger than Evans’s focus group. These participants also articulate “victimhood” narratives and contrast themselves with their mothers. However, their distinct position as the only daughters and their struggles with intense body-focused anxiety were not present in Evans’s ethnography. This points to a possible shift from a gendered subject narrative to a more biopolitical form of enculturation and gendered awareness. In both Evans’s study and my ethnographic data, both mothers and daughters repeatedly highlight the unique situation of having or being the only child and the only daughter. I argue that both intergenerational changes in gender awareness and women’s bonds and contrasts with their mothers need to be understood within the context of the transformation of Chinese family structure.

## Intergenerational Trauma and Historical Transitions

Few ethnographers and mental health professionals have examined the transmission of intergenerational trauma and its effects on mother–daughter relationships within patriarchal families (Mason, 2020). Intergenerational trauma has been explored across various disciplines, including clinical settings, social sciences, history, and cultural studies (Davoine & Gaudillière, 2013; Noormohamed, 2012; Prager, 2003; Walkerdine et al., 2013). The examination of intergenerational trauma originated from research on Holocaust survivors, shedding light on how long-repressed memories can result in challenges in forming connections and distorted perceptions during stressful times (Connolly, 2011; Davoine & Gaudillière, 2013; Kidron, 2003; van der Kolk, 1994; Walters et al., 2011). Children of trauma survivors frequently exhibit dysfunctional traits, including trouble distinguishing between reality and fantasy, especially concerning perceived threats, as well as disruptions in the perception of time, which hinder identity development (Connolly, 2011).

In the Chinese context, several unique cultural factors contribute to the transmission of trauma across generations. Between 2018 and 2019, I conducted ethnographic research in various Chinese cities, focusing on the lived experiences surrounding personal and historical transitions among different generational cohorts. Through person-centered interviews and participant observation with forty individuals aged 19 to 70, I utilized phenomenology as my philosophical foundation and methodology (Csordas, 2002). This approach enabled me to examine how individuals perceive and experience personal and social transitions in their bodies.

Embodiment serves as a foundation for analyzing body–world interaction, enabling a broader interpretation that transcends observable events and embraces the dynamics of macro-level social changes while considering micro-level interactions (Csordas, 2002). By contextualizing individuals’ lived experiences within family units, I traced life trajectories and examined how people embody radical transitions through everyday interactions (Csordas, 2002). The social turmoil affecting my participants included significant events such as the Japanese invasion (1937–1945), the civil war (1945–1949), the Great Leap Forward (1958–1963), the 3-Year Natural Disaster Famine (1959–1961), the Cultural Revolution (1966–1976), and the socioeconomic upheaval during market reforms, which led to the dislocation of communities and transformations in kinship systems (Markert, 2018; Peng, 1987; Rofel, 1999; Zhou & Hou, 1999).

My observations identified three factors related to an increased risk of intergenerational trauma amid social turmoil within a family:

- **Precariousness from Social Transitions:** Rapid changes in social structure create a sense of precariousness across historical periods, prompting each generational cohort to seek security while navigating morally and politically ambiguous ideologies and norms. This serves as the social backdrop of why many Chinese people nowadays constantly experience anxiety as they go through life stages, afraid of being “left behind” when the next big change in social policy would create more disaster than opportunities (Kleinman et al., 2011; Zhang, 2020).
- **Multiple Levels of Trauma:** Trauma affects families and individuals on multiple levels. While social turmoil is a common experience, those directly subjected to physical violence—both domestic abuse and political persecution—are more likely to carry trauma and transmit it to future generations through insecure attachment and difficulties in intimate relationships. This theme is evident in my family observations, where mothers who suffered from adverse childhood experiences are much more likely to struggle with mental illnesses and have challenges maintaining nurturing and supportive relationships with their own daughters.
- **Lack of Discourse on Trauma:** Intergenerational trauma is more prevalent in families where previous generations lack access to public dialogue about their experiences. Without socially recognizable discussion or acknowledgment, dysfunctional social relations and outdated coping strategies can lead to feelings of being “stuck.” These experiences result in the subject’s repeated emotional turmoil and often demonstrate little trust, even paranoia, in social interactions (Author, 2021). This feature is most pronounced when mothers unintentionally express resentment toward their daughters or spend excessive time recounting their early struggles, which their daughters have been forced to confront from a young age.

## Becoming Chinese Daughters: The Hauntology of Son-Preference Culture

Women and girls in China are victims of enduring misogynistic structures, gender injustices, and violence in both public and domestic spheres. Research indicates that son preference remains a critical ideology in many families, despite cultural transitions and gender equity movements since the establishment of the People’s Republic of China (Aghatie & Gangoli, 2015; Croll, 2001). The son-preference culture emphasizes the significance of sons as core family members and carriers of family names, leading to greater economic and emotional investment in sons compared to daughters. This dynamic persists even in families with only one child (Watkins et al., 2010). Daughters are often regarded as temporary “tenants” of the family, expected to provide obedience and support while simultaneously being marginalized (Croll, 2001; Won Kim & Fong, 2014; You, 1990).

The one-child policy, in effect for 35 years and lifted on January 1, 2016, has significantly impacted the economic, demographic, familial, and moral landscapes of China (Ebenstein, 2010; W. Feng et al., 2016; White, 1994). The influence of this policy on the experiences of both mothers and daughters is complex. Some studies indicate that the policy has empowered urban daughters by providing them with undivided emotional and economic investment (Fong, 2002). Being the only daughter in a Chinese family is an unprecedented situation in Chinese history. Conversely, other research highlights how the policy exacerbated a son-preference culture, leading to practices such as gender-based abortion, female infanticide, and the adoption of daughters (Croll, 2001; Ebenstein, 2010). As the policy has been lifted, parental investment in daughters is increasingly influenced by the presence of younger siblings and differences between urban and rural areas. Children report varying subjective perceptions of treatment based on gender (Hu & Shi, 2020). These findings suggest that the experiences of Chinese daughters are heavily shaped by population policies and are inherently complex.

While the one-child policy may have empowered urban daughters, the trauma endured by their parents continues to haunt their lives, despite increased economic affluence (Mason, 2020). One participant described her impression of “the half-heartedly suppressed story about gender-selection adoption or infanticide” in her family as an “East Asian horror story that haunts me in ways I can’t articulate.” “My grandparents aborted a girl and then sent my mother away for most of her life. When my mother recounts this story, her narration sometimes shifts from ‘I’ to ‘we’, as if she and her unborn sister were one, and the feelings of abandonment and abuse were intertwined. When I was born, my mother felt both glad and disappointed because I was a girl.” When I asked my mother why she experienced such conflicting emotions upon learning her daughter’s gender, she replied, “Because we were going to go through the same thing again. How many of us does it take to make up for a son?”

Confused about who exactly “we” were in those contexts, I eventually understood that the haunting trauma of “not being a son” was far from resolved by the One Child Policy. Each singleton daughter unconditionally bears the weight of not only her surviving mother’s fate but also that of her unborn or abandoned aunts and sisters. Hauntology, rooted in Derrida’s theories and psychoanalytical frameworks, investigates the complex interplay of trauma within individuals’ lived experiences. It reveals how traumatic experiences can be both hidden and visible throughout personal life trajectories and historical transitions (Good, 2019). In my case studies, what haunts survivors and their descendants is the deprivation of dignity based on birth gender, compounded by historical trauma and social upheaval. This trauma is often too immense to confront without therapeutic assistance, leading to silence and limited discussion in public spaces (Author, 2021; Micale & Pols, 2021). Simply saying “son-preference culture” is a historical relic and erasing it from the discourse is part of the structural violence in current society. Gender-based violence is frequently normalized within a patriarchal context, leaving victims without a means to articulate their suffering, especially when the perpetrators are family members (Lamela & Figueiredo, 2013; Mason, 2020; Sangren, 2017). Across many families, I studied, “being” a son “naturally” came with social responsibilities and positions. However, “becoming” a daughter often entails a lifelong struggle with conflicting ideologies and repressed intergenerational trauma that originated long before they were born.

## Method

My ethnographic research, conducted periodically between 2018 and 2025, focuses on the embodiment and emotional experiences across historical and personal transitions. Over 7 years, I have organized the ethnographic and survey data from 25 young women across three studies (intergenerational trauma, COVID-19 pandemic mental health study, and negative emotion and human sociality study). As the concept of body-focused anxiety manifests, I centered my inquiry on mother–daughter relationships, developing interview questions to explore this dynamic. From 2020 to 2022, I conducted online interviews, followed by additional rounds of follow-up interviews and participant observations in 2023 and 2024. This approach fostered long-term relationships that enriched the data collection process.

The ethnography included long-term case studies of 25 young women, aged 19 to 22, recruited from 2018 to 2022. Of these participants, 24 were only daughters, while one had a sister 13 years younger. I traveled and met with them online across seven cities and towns, covering most of China’s first- and second-tier cities. Their socioeconomic backgrounds varied, ranging from lower-middle class (with family incomes around 50,000 yuan per year) to upper class (with families owning multiple businesses). Qualitative data, including daily diaries and routine traces, were primarily collected in 2021, 2022, and 2024.

The strained relationship between mothers and daughters has posed challenges for me, as a member of the post-80s generation, in engaging with the mothers of these young women. Despite limited conversations, most mothers expressed significant worry and helplessness regarding their daughters’ fragile mental and physical states. “Becoming a mother of a sick daughter” has emerged as their new core anxiety. The desire for their daughters’ recovery often creates rigidity in the mother–daughter relationship, as mothers grapple with guilt and confusion, uncertain about which of their behaviors may have contributed to their daughters’ ongoing symptoms. The more they attempt to engage in their daughters’ lives, the more friction arises. As my relationship with my participants deepened, some allowed and even encouraged me to interact with their families and mothers, while others explicitly prevented such interactions, fearing it could be triggering. One participant explained, “My friendship with you is one of the few social relations where I feel no stress or shame. I kind of do not want my family to interfere with that.” On other occasions, daughters were eager for me to meet their mothers, but the mothers were reluctant, fearing judgment as bad mothers, knowing their daughters had become my acquaintances due to mental health struggles. Consequently, my research data primarily reflect the daughters’ perspectives, and I will present their side of the story in this paper.

To support my argument that intergenerational trauma significantly contributes to body-focused anxiety, I organized observations and ethnographic data around several key themes: types of body-focused anxiety; who are influential figures in their lives; major life-trajectory narratives (mainly interviews and diaries); daily routines and reflections (mainly from semi-structured interviews, weekly check-in journals, and participant observations). 10 participants encouraged me to meet their mothers, while the other 15 actively refused to introduce me to their families for various reasons. In this article, I center the daughter’s experiences while also incorporating mothers’ perspectives, drawn mainly from conversations or observations of their interactions with their daughters. I will now illustrate this article with three case studies to demonstrate how the mother–daughter relationship and intergenerational trauma significantly influence body-focused anxiety. These cases were selected for several reasons. First, none of the women in these studies were officially diagnosed with eating disorders or body dysmorphia. Second, they all experienced varying degrees of mental health issues. Third, their mother–daughter narratives are the most representative of all 25 cases, and their articulations provide deep insight. Fourth, as my long-term participants, their involvement in interviews, observations, and surveys offers comprehensive data across different stages of their lives.

## Marie: Haunting “Imperfections”

The first time I had an in-depth conversation with Marie about her body was during the COVID-19 lockdown. At that time, Marie was 21 years old, had graduated from a prestigious university, was working at her first job, and had been accepted into graduate school. In her later recollections, she noted that she “didn’t like the job, didn’t understand why she was pursuing graduate studies; perhaps it was due to a period of confusion and a specific fear of wasting time, especially concerning her mother’s comments about it. So, while her body and energy were extremely exhausted, she continued doing things she disliked.” One day during the lockdown, she received a pile of tasks that overwhelmed her, leading to a sudden loss of appetite. Although we had discussed her health struggles several times before, that online meeting was prompted by Marie having gone nearly 72 h without eating, unable to go out, and struggling to get out of bed. She had entered a state of stagnant despair.

“The lockdown was just the last straw,” she told me during the interview. “I first started feeling like everything had stopped when I was seven.”

At the age of fourteen, Marie jumped from the fourth floor because she could no longer endure her mother’s strict discipline and frequent “unpredictable temper and sudden accusations.” She broke both legs and seemed to have injured her lumbar spine as well. “In total, I underwent a dozen surgeries that I remember no details at all, and now there are various kinds of screws and nails in my body. I did not understand what happened.” When she recalled this event, Marie sincerely told me, “Because of the surgeries, I was bedridden for almost 3 months. That was the best memory of my adolescence.”

“Why do you say that?” I was astonished.

“Because I could finally take a break from school.”

In Marie’s recollections, her suicide attempt did not significantly disrupt her already stressful life. Her mother could not face her suicidal attempt, insisting that Marie not disclose it. In their small town, she publicly maintained that Marie had accidentally fallen from the balcony, viewing her daughter’s suicidal thoughts as a source of shame. While Marie recovered in the hospital, her mother arranged for teachers to tutor her, fearing she would fall behind. These tutors told Marie that threatening suicide was irrational and unfilial. Marie returned to school without rehabilitation, which led to the atrophy of her calf and back muscles and a decline in her physical abilities.

In high school, academic pressure and the emotional strain with her mother worsened her condition. Marie developed polycystic ovary syndrome, experienced irregular blood sugar and menstrual cycles, and began gaining weight. Her mother’s anxiety escalated as she fixated on Marie’s menstrual cycles and body shape, believing they would affect her future reproductive health.

In 2020, Marie was diagnosed with depression, leading her to quit her job and pause her graduate studies. From 2020 to 2023, she experienced severe agoraphobia, struggling to meet friends or eat out. Her daily routine collapsed, and managing her appetite, sleep, and emotions became increasingly challenging. During this time of isolation, her mother’s visits to Shanghai often triggered profound stress and trauma for Marie, culminating in episodes of insomnia and erratic eating patterns. Marie’s relationship with her mother was filled with conflicts and opposition. During Marie’s extremely harsh childhood, her mother would occasionally recount being one of four daughters in a family without sons in their small town. She believed she had transcended the class of her original family through personal effort and used the threat of “if you don’t study hard, you’ll have to go work the fields or beg in the countryside. I’ll disown you for being such a shame.”

Marie expressed gratitude for her existence, sharing how her mother faced immense pressure regarding her pregnancy. Initially, when her mother discovered she was pregnant with a girl, she was compelled to abort under her grandmother’s intense pressure and her father’s silent compliance. Marie highlights her mother’s pain, recalling the nights of crying after the abortion, with her hair stuck to the pillow. For Marie, her mother’s decision to carry her to term was a testament to the value of her life. “I owe her my life,” Marie once stated, “yet I’m not sure how I can repay this debt while resenting the harm she inflicted on me. I feel wronged and my stomach hurts when she accused me of being undaughterly.” Filial piety, as it transforms into everyday moral emotion, is deeply rooted in a sense of indebtedness and guilt.

In my research of Chinese mother–daughter relationships, a profound and contradictory emotion persists: daughters perceive their mothers as both their greatest source of pressure and their deepest source of loyalty. This dynamic becomes even more complex as daughters enter womanhood. Following the one-child policy, sex-selective abortions remain prevalent, even in developed regions like Jiangsu and Zhejiang. Many mothers, forced to abort or give away baby girls, often feel they have not achieved the expected outcome of bearing a son. This leads to a sense of helplessness as they confront what they perceive as their failures—giving birth to daughters instead of becoming mothers of sons. These feelings create dual identities as both failed daughters and failed mothers, trapping them in an existential crisis, with the haunting memory of the “missing sister” looming over the only daughters of the new era. The notion of “failed gender” has not been included in the discourse. Parents often state, “Having sons or daughters is the same” in millennial China. However, many admit that son-preference culture is still prevalent in families and everyday life. The unaddressed trauma continues to be passed down in the form of “becoming a daughter that is better than a son.” In my interviews, many mothers, like Marie’s mother, place great “concerns” on their daughters. This concern is driven by deeply harbored resentment, stemming from both perceived injustice and heightened expectations for their daughters to perform better because “now there is no other.” This expectation for daughters to surpass sons is reinforced through their mothers’ ongoing “transformation” of their daughters according to standards that are often unclear, even to themselves. Abused mothers who did not give birth to sons, despite having different professions and belonging to various social classes, reflect a unique temperament shaped by the market economy boom of the 1990s and the rise of feminism: to strive, to work hard, and to be competitive. Often, this response aligns with a positive social atmosphere (Rofel, 1999), but the unresolved trauma of “not being or having a son” transforms this positive pursuit into compulsive perfectionism. In the mother–daughter relationship, this compulsive perfectionism manifests in three ways: the continuous modification of their daughters’ bodies, the demand for excellent academic performance, and the exhibition of “cleanliness” in physiological, moral, and social aspects.

In Marie’s narrative, a striking comparison arises: “My rental apartment is often so messy I can barely find a place to stand. Besides the depression draining my energy, I sense it might be a neurotic resistance. When I return to my mother’s house, I feel there is no space for my existence. She is obsessively clean, yet her environment lacks comfort. For instance, she invested in expensive mahogany furniture but scolded me for putting my books and laptop on it, fearing I would cause damage. Ironically, she meticulously wipes the mahogany table, worrying about cleanliness, and eventually really damages it. To this day, when I hear furniture squeak on the floor, my heart misses a beat, fearing I will be yelled at.” Subconsciously, it seems Marie uses her physical space to set boundaries for herself, reflecting her illness in her environment. In 2025, unable to cope with her psychological state, Marie gave up her graduate thesis. “I finally don’t have to study anymore,” she texted me after sleeping for nearly 16 h. The following week, her menstrual cycle resumed. In response, her mother voiced concerns about her daughter’s future employment while stating that she respected Marie’s decision. One month later, however, their relationship became turbulent again when her mother’s friends asked about Marie’s career. Feeling ashamed about her daughter, her mother once again lashed out at Marie.

### Cynthia: Continuous Body “Reconstruction”

When Cynthia started participating in interviews in 2022, she was 25 years old and pursuing a doctorate. She expressed, “I have serious body issues.” When I asked for clarification, she elaborated, “I deal with irregular eating patterns—sometimes I starve myself or binge eat emotionally. My relationship with pain complicates things; I often hurt my body when I’m stressed.”

Cynthia mentioned that her struggles with body image began way before puberty, but she couldn’t remember exactly when. “My mom always criticized me for being fat and not looking good,” she said. Throughout her story, Cynthia often linked her body issues with what she called “Mommy Issues,” sometimes subconsciously and other times consciously. They experienced many body transformations together, leading to significant conflict and deep connection.

Cynthia first attended a weight loss summer camp at just 8 years old. She was the only child there without adult supervision, surrounded by adult participants and teens. “Looking back, that weight loss camp employed many pseudoscientific and inhumane methods. I can’t believe they were allowed to operate,” she reflected.

“Why would you send an eight-year-old to a weight loss camp? Were you overweight then?” I inquired.

Cynthia hesitated, saying, “My mom thought I was fat. Maybe I was just chubby. I don’t know. I wasn’t clinically overweight.”

At the camp, Cynthia was forced to try numerous weight loss techniques. These included fasting, drinking herbal tea, taking medication, and being struck with a paddle. “They claimed it would boost my blood circulation and metabolism. But I ended up bruised all over and in severe pain. I also underwent daily cupping therapy, which left burns on my skin.” When she returned home, her mother was not upset about what Cynthia had endured; she was disappointed that Cynthia hadn’t lost any visible weight.

During middle school, academic pressure and bullying worsened Cynthia’s body image issues. Together, she and her mother tried extreme weight loss methods, such as consuming only a small piece of boiled beef daily and taking long hot baths to burn calories. In just 4 months, Cynthia lost thirty kilograms. However, she began feeling dizzy when walking, and her blood sugar levels became unstable. Hunger started to symbolize self-control and discipline; escaping the cycle of starving herself became difficult unless her blood sugar fell drastically, leading to an emotional breakdown.

Even years later, as Cynthia’s doctoral studies intensified, these behaviors occasionally resurfaced. When the pandemic struck, our conversations moved online. Despite the high travel costs between China and the U. S., Cynthia’s mother managed to bring her back to China during the lockdown. She insisted it was necessary because she believed her daughter “looked bad from a certain angle in photos,” pushing her to have cosmetic surgery. Although Cynthia found this absurd, she complied. It seemed she would resist this mother–daughter body reconstruction in theory but would participate behaviorally, caught in a cycle of shared body image issues. Cynthia’s mother, Ms. Yang, was born into a wealthy family in southern China. As the middle daughter, she was seen as “another failed attempt” by her grandmother to have a son after several failures. At a young age, she was sent to live with her aunt. Although her aunt treated her well, she often felt like a burden. Her cousin viewed her presence as an invasion, frequently adopting a hostile attitude toward her. It wasn’t until her teenage years that she returned to her parents’ home. She had a distant relationship with her parents and a close yet competitive dynamic with her sisters. In Cynthia’s view, her mother was a strong-willed woman. As a teenager, she traveled to Hong Kong to buy designer brands to enhance her appearance, becoming one of China’s first generations of “it girls” in the market era. In her career, she strived for success, even purchasing more expensive cars than her boss to demonstrate her professional capabilities. Having given birth to a daughter, which seemed like a “failure” in a southern Chinese family that valued male heirs, she sought to make her daughter more competitive than any son could be—more outstanding, more exceptional, and more beautiful. Ms. Yang actively participated in her daughter’s continuous transformation, sending her to better schools to hone her competitive edge and encouraging beauty treatments, weight loss, and body shaping from Cynthia’s elementary school years.

As Cynthia deepened her understanding of social science and feminist theory, she began to critically and dialectically view this “war that could never be won,” pointing out that the real bullies in her life were her father and paternal grandmother, who stood against her mother as the embodiment of the unjust patriarchal family structure. This dialectical perspective allowed her to empathize with and understand her mother better, but it also made achieving bodily autonomy from her even more challenging. When Cynthia felt her mother’s pressure, she exhibited loyalty and a desire to rescue her. She would reject her mother’s interference while simultaneously deepening her body reconstruction. For instance, she found it ridiculous that her mother would go to such lengths to send her weight loss meal replacements from China to her home in the U.S. Yet, every time such incidents occurred, she would unconsciously start dieting or begin a new, more intense gym routine. Not uncommon among some young women from the upper middle class, Cynthia had an alternative interpretation of wealth, consumerism, and self-presentation compared to her mother. However, when such ideological conflicts arose, she sometimes directed the embodied stress onto another round of body reconstruction to ease the emotional tension from diverging paths with her mother. “Understanding the reasons behind things rationally, even the systemic injustices, doesn’t alleviate my body anxiety. Especially since this entire philosophical reflection involves not just me; my mother is also entangled in it. Sometimes, I see my struggle with body issues and my mommy issues as more of a coping strategy against patriarchy.” As one of my long-term interlocutors, Cynthia’s ongoing ‘body modification’ reflects the fluctuations of life pressures and shifting dynamics of her intimate relationships. Like Cynthia, many young women experience body-focused anxiety in constant dialogue with their mother–daughter relationships, self-understandings, and other intimacies, all shaped by and pushing back against the enduring influence of Chinese patriarchy. The ways they navigate their body-focused anxiety are never linear.

### Echo: An Exodus from My Mother’s Gaze

At 27, Echo decided to reverse her cosmetic surgery. “I didn’t do much—just some cheek enhancements, jawline sculpting, double eyelid surgery, and a nose job. But now I’ve decided to remove all of it.”

During our video chat, Echo specifically told me that she would turn off all filters and show me her face without any makeup. Although she had just undergone a series of surgeries, she was glowing; even her skin seemed to have improved. This relieved me. Before Echo decided to undergo this series of reversal surgeries, she had experienced about 2–3 months of disordered eating. In our earlier meetings, she was rapidly losing weight, dealing with bodily inflammation, and enduring continuous low-grade fevers.

Echo was one of my earliest core interview subjects. When she joined my field study in 2018, she told me, “I probably have every common mental illness you can think of, so you can gather a lot of data from me.” Among the “series of common mental illnesses diagnoses” she mentioned, the most impactful on her daily life and physical condition were Complex PTSD, along with accompanying somatic pain and emotional dissociation. These two symptoms mirrored each other alongside her disordered eating and insomnia. During the worst episodes, Echo would swallow up to 16 painkillers a day. “Later, I realized I couldn’t continue like this,” Echo said. “I had to consciously learn how to engage with my body and how to live my life.” During her best days, Echo was a foodie. She often explored complex recipes, created exquisite dishes, and shared them with friends. However, when her condition began to decline, she would experience memory lapses, sudden fainting spells, and forget when she last ate. In the brief moments when her somatic pain subsided, she would uncontrollably rush to the bathroom to induce vomiting. Between 2018 and 2023, due to emotional dissociation, Echo was the only patient unable to provide me with a complete narrative of her life story. When repressed memories resurfaced, she struggled to confirm whether they were indeed her own experiences.

The chaotic self-perception and somatic symptoms she experienced stemmed from Echo’s tumultuous childhood. Echo described her mother, Ms. Zhang, as “the eldest daughter in the family,” embodying the archetype of the typical eldest daughter in a Chinese family. “She was abused by my grandfather from a young age, and all the family’s resources were naturally directed toward my uncle, her brother. My grandfather is the type of father who would slap my mother in the face, even today, without hesitation. My uncle, pampered but lacking proper education, is a gambling addict who has lost the family’s fortune yet still expects my mother to support him.” Growing up in such a violent household, Ms. Zhang’s marriage ended shortly after it began, marred by emotional and physical violence. Both parents refused to raise Echo, who grew up under the care of her abusive grandfather and submissive grandmother, often being used by her parents to manipulate each other. Although there is no complete narrative, Echo’s experiences of fainting, somatization, and memory lapses often followed her parents’ family disputes. These memory repressions and dissociations frequently manifested as anorexia or alcoholism in her early adulthood.

“My mother is a very beautiful woman,” Echo told me. “My stepmother is as well. In this regard, I believe my dad is proud of his taste. He was the first to suggest that I undergo cosmetic surgery, which is one of the few areas where my parents reached an uncommon consensus in my life. They both seemed to want the people around them to be beautiful enough not to embarrass them.”

Echo’s cosmetic surgery journey began at 18. Although many surgeons refused her due to her naturally narrow nasal cavity and jaw misalignment, Ms. Zhang went to great lengths to find a private hospital willing to perform the surgery for a high price. Throughout the process, Ms. Zhang continued to examine her daughter’s face from every angle possible, commenting on how she should present herself. Echo described it as her “mother’s gaze,” a highly critical judgment that was a natural part of her mother’s social interactions and cognitive processes. “Do you understand my mother’s gaze now?” Echo asked me after one of our gatherings. “Please don’t take it personally; she’s not targeting you. She gives that kind of X-ray-like scrutiny to all my friends. She can’t help it.”

After experiencing somatic pain, anorexia, depression, interpersonal crises, career pressures, and renewed neglect and betrayal from family members, Echo suddenly identified the root of her sadness. “It’s my nose job,” she told me. “I’m not speaking metaphorically. I genuinely believe that the heart of my sadness lies in my nose. The other day, I saw a childhood photo of myself, and I felt that my face reflected a very natural state, with features that were not wrong and a natural joy. My body might perceive having surgery due to my mother’s anxiety as a betrayal, so I decided to remove it.”

Before this outburst of somatic pain and the subsequent decision to reverse her surgery, Echo recalled during therapy and family discussions that she had been sexually molested by her cousin when she was very young. She brought this up with her mother, seeking either comfort, confirmation, or recognition of the trauma stemming from her childhood abandonment. “Why can you see so many problems with my body but not realize that I needed help?” Unable to confront such questions, Echo’s mother remained silent. It took several weeks for her dismissal to sink in and become hurtful to Echo. According to my record, Echo’s memory over this issue began to scatter again, and her health eventually declined in the following several months. After she “removed the surgery that symbolized her sadness,” she once again forgot about this resurfaced memory. It was as if she was processing trauma entirely through her body, the only entity over which she currently had total control. When I last discussed the state of Echo’s mother–daughter relationship, she expressed that she was increasingly aware of the violence her strong and beautiful mother had endured and the anxiety that had built up within her without relief. “But I need first to find a way to deal with my issues and, in some ways, accept that her gaze and criticism are also her way of coping with herself.”

## Dealing with the Mother–Daughter Intersubjectivity

In tracking the life courses of 25 young women over the past 7 years, 19 reported experiencing aversive childhood situations or significant emotional or physical abuse, with 16 identifying their mothers as the primary source of stress. Although this sample may not be statistically representative and is potentially self-selective, focusing on women sensitive to body-focused anxiety reveals crucial insights. This anxiety highlights the need to examine its roots through intergenerational trauma, which I define within the context of gender-based violence perpetuated by a patriarchal system. This trauma is transmitted through the formation of an intersubjective bond between mothers and daughters, where unresolved existential tension stemming from psychological abuse suffered by the mothers is projected onto their daughters. These case studies reveal how mothers, once unwanted daughters, attempt to fix their existential crises by transforming their daughters’ bodies. The daughters navigate a complex mix of emotions—love, neglect, jealousy, devotion, resentment, and aspiration—without cultural discourse to understand the mother–daughter dynamic. They embody an existential crisis, feeling compelled to transform and correct a perceived wrongdoing: their mothers’ regret of not being born a son. Intergenerational trauma is thus perpetuated through the mother–daughter intersubjectivity, as each generation confronts gender bias within a patriarchal structure amid cultural transition. Many young women I interviewed are troubled by this intergenerational trauma, which is not widely recognized in Chinese social sciences outside therapy settings. Trauma from gender-based violence in previous generations manifests in the next as severe body-focused anxiety, gender anxieties, and somatic symptoms, often concealed by overt clinical diagnoses. Although few mothers explicitly expressed gender preference, they all held high expectations for their daughters while reflecting on their own unjust treatment as daughters. The pressure to “become” a daughter more capable than a son, intended as compensation for maternal traumas, significantly influences their upbringing, yet rarely fosters true healing.

One significant characteristic of intergenerational trauma-influenced “body issues” is that mothers perceive their daughters as extensions of themselves. The recognition they sought during their own childhoods—validation for their bodies and gender—was projected onto their daughters. In a casual conversation, one mother inadvertently remarked, “I really had a harder time than she did when I was young... what did she do to deserve being treated so well?” Many mothers from the 60s and 70s revealed an unspoken jealousy toward their only daughters, who lead materially superior lives. Their frequent emphasis on their own hardships whenever their daughters enjoy material affluence sometimes implies such feelings. This jealousy, which contradicts the ideal maternal image, complicates mothers’ ability to confront shared traumas with their daughters. On a conscious level, some mothers genuinely believe that their efforts to “correct” their daughters’ bodies and body–world interactions are part of their maternal duty, unaware that such interventions can alter their daughters’ self-perception and even introduce internalized shame and gendered suppression throughout the process of womanhood.

Additionally, fathers were largely absent in these narratives. In my research, fathers were often invisible, participating minimally in their daughters’ lives. Within the transforming patriarchy, fathers were largely clueless about how to be a father to a singleton daughter. In the worst scenarios, they become the source of conflict in the mother–daughter dynamic. Mothers either confront their husbands or feel jealous of the simpler relationship between fathers and daughters, “forcing me to appear like a villain.” The impact of fathers will be another topic for further research.

## Conclusion

The case studies illustrate that young women’s body-focused anxiety extends beyond anorexia and binge eating to encompass emotional dissociation, somatization, disrupted daily routines, and challenges in organizing self-perception, altogether constituting a series of crises women face in relation to their bodies. These anxieties are closely tied to intergenerational trauma, as mothers often view their daughters’ bodies as extensions of their own, projecting expectations and regrets that perpetuate boundary-less mother–daughter relationships. Over the past 7 years, however, these relationships have shown gradual improvement: daughters remain empathetic while also asserting boundaries, signaling a shift influenced by feminist movements and heightened post-2020 feminist awareness. Many mother–daughter relationships are now beginning to move beyond trauma narratives, opening space for more philosophical discussions about embodiment, autonomy, and intergenerational care.

This paper highlights the experiences of body-focused anxiety among women in certain urban areas of China. I believe intergenerational trauma significantly influences the somatic symptoms experienced by women born in the 1980s and 1990s. The violence and trauma faced by their mothers are transmitted to their daughters as anxiety about becoming women, manifesting as a pursuit of perfection and ongoing body modifications. The daughters’ traumas often begin before birth, leading to challenges in integrating their self-perceptions due to harsh treatment and excessive expectations. I hope this research provides insights into the social conditions of women in China and East Asia, the emergence of body-focused anxiety, and the processes of intergenerational trauma. Chinese women should not be viewed as a homogenous group, and the complex nature of mother–daughter relationships deserves further exploration. In today’s evolving society, detailed depictions of various female relationships are essential for understanding how each generation’s experience of “becoming a woman” specifically impacts everybody and transformation.

## Data Availability

No datasets were generated or analyzed during the current study.

## Footnotes

1 The collectivist era in China spans from the establishment of the People’s Republic of China in 1949 to the 1980s. Due to the Open Door Policy in 1978, scholars usually indicate the collectivist era eventually dissolved with the end of planned economy and with other social structural and cultural transformation. The collectivist era was characterized by the prioritization of collective over individual interest, with policies aimed at fostering communal living and economic practices. The era saw the implementation of land reforms, collectivization of agriculture, and the formation of communes and production teams. Yan Yunxiang describes this era as one where individual autonomy was submerged under collective goals (Yan 1998), highlighting the state’s emphasis on social unity and economic equality. The Great Leap Forward (followed by the biggest famine in the twentieth century) and the Cultural Revolution (1966–1976) further reinforced collectivist ideals with detrimental consequences and deeply traumatize the Chinese population.

2 The One Child Policy, introduced in China in 1979, was a government initiative aimed at curbing the nation’s rapidly growing population to ensure economic stability and resource management. Under this policy, urban couples were generally restricted to having only one child, with exceptions for rural families (if the first-born was a girl, another child was allowed after 6 years) and ethnic minorities (no restriction). Susan Greenhalgh notes that the policy was a radical social experiment designed to transform the demographic landscape in China (Greenhalgh, 2003). It led to significant social and cultural impacts, including changes in family structure and gender dynamics, as families often favored male offspring. Some research indicated a surge in gender-selected abortion, infanticide, and daughter-abandonment (Croll, 2001). Other research showed an increased appreciation and equal opportunity for daughters, especially in urban China (Zhang & Fong 2014). While the policy contributed to the reduction of birth rates, it also resulted in unintended consequences such as gender imbalance and aging population. In 2016, China officially ended the policy, allowing families to have two children.
